# Till the Doctor Comes

**Published:** 1886-10-09

**Authors:** 


### Till the Doctor Comes.

[Inquiries and Communications for this column should be addressed, "Emergencies," care of the Editor of The Hospital.]

It is desired to leave matters of treatment to the Medical press, but this column of The Hospital may usefully contain advice to mothers, nurses, and others, as to how to act in certain emergencies, until the doctor comes. Many practical hints on nursing cannot fail to prove helpful to our readers. Much that appears in this column will be extracted, with the permission of the author, from two little books for family use published by Messrs. Kegan Paul, Trench, and Co., entitled, *Help in Sickness*, and *Helps to Health*. Of the former of these books, the *Lancet* declares: "We have often desired to obtain, either for ourselves or friends, the very information this book supplies. Indeed, we feel that no medical or general library can be complete without such a book of reference."

### I.—DISEASES OF CHILDREN.

The diseases to which children are subject are, as a rule, those which attack older people; but there are undoubtedly diseases peculiar to children; and diseases in children are so modified by the marked excitability of their nervous system and other peculiarities, that something, however little, must be said concerning them. It is impossible here, however, to go into the subject fully. To do so would alone more than fill the space allotted to this section of The Hospital. Now children can tell us nothing; all their symptoms must be made out by careful observation of their ways, their appearance, their manner, etc., and the utmost must be made of the least change from the child's habitual demeanour. After some experience it is easy for a medical man to tell almost with certainty, from simple observation of a child for a few minutes, what that child may be suffering from; and this habit of observation must be acquired by anyone wishing to become a good nurse, or to be useful to children in their various maladies.

**A Child with Inflammation.** As an example, take the case of a child with inflammation of the lungs or chest walls. He will probably be found lying on one side, and will show repugnance if an attempt is made to turn him on the other; the breathing will be very hurried, and the breaths short and shallow; he will have a short, dry cough, interrupted almost as soon as it has commenced; there will be no wheezing and rattling, as in bronchitis; he will spit nothing up at any time, but this is peculiar to children, as they swallow the phlegm at once, and cannot be taught to spit matters out of their mouth; the face will look flushed and feverish, and the nostrils will be hard at work with each breath; the pulse will be very fast; the body will feel extremely hot and dry, seeming to almost burn the hand; the cry will be like the cough, merely a short, sharp whimper, interrupted almost as soon as commenced, as if the attempt gave him pain; his whole time will be taken up, as it were, with the difficult task of breathing; he will wish to be left alone, and probably show repugnance to any interference, declining all food, except cold water, which he will drink with avidity.

**A Contrast.** Much more might be pointed out as to the different stages of the disease, but, as a contrast, let us take bronchitis, and show the differences between the two. Here the little sufferer will still breathe quickly, and the nostrils will work rapidly, more so as the disease progresses; there will obviously be much secretion about the lungs, and wheezing, rattling noises will be heard in the chest and throat; the cough will be much longer and looser; it will evidently be attended with much expectoration, which is usually at once swallowed, but occasionally it will come up in quantities, and can be wiped out of the mouth; he will not lie on his side, but will like to be raised somewhat in bed, as he can then get his breath more easily; the skin will not feel hot and dry, but be covered with perspiration, brought on by the muscular exertion of working hard for his breath; the pulse will be fast, but perhaps chiefly from the exertion of breathing, much force being required, as it were, to pump the air in and out of the obstructed tubes, and food will still be declined. This will show what can be done in the matter of observation in children, and each different disease might be taken *seriatim*, and the differences noted; enough, however, has been given for an example. It will be the nurse's duty to note all these points, however trifling, and report them to the doctor. A few of the more common ailments of children and their treatment will next be shortly described.

(*To be continued.*)

